# Clinical Outcome Patterns of Use of Radium-223 in Patients with Metastatic Castration-Resistant Prostate Cancer

**DOI:** 10.3390/curroncol31110480

**Published:** 2024-10-22

**Authors:** Colleen Mackenzie, Jasna Deluce, Morgan Black, Emma Churchman, Eric Winquist, Scott Ernst, David T. Laidley, Matthew Parezanovic, Kylea Potvin, Ricardo Fernandes

**Affiliations:** 1 Schulich School of Medicine and Dentistry, Western University, London, ON N6A 3K7, Canada; colleen.mackenzie@lhsc.on.ca; 2 BC Cancer—Abbotsford, Abbotsford, BC V2S 0C2, Canada; jasna.deluce@bccancer.bc.ca; 3 Verspeeten Family Cancer Centre, Victoria Hospital, London Health Sciences Centre, London, ON N6A 5W9, Canada; morgan.black@lhsc.on.ca (M.B.); emma.churchman@lhsc.on.ca (E.C.); eric.winquist@lhsc.on.ca (E.W.); scott.ernst@lhsc.on.ca (S.E.); matthew.parezanovic@lhsc.on.ca (M.P.); kylea.potvin@lhsc.on.ca (K.P.); 4 Division of Medical Oncology, Department of Oncology, Schulich School of Medicine & Dentistry, Western University, London, ON N6A 5W9, Canada; 5 Medical Imaging Department, Division of Nuclear Medicine, Victoria Hospital, London Health Sciences Centre, Western University, London, ON N6A 3K7, Canada; david.laidley@lhsc.on.ca

**Keywords:** castration-resistant prostate cancer, radium-223, clinical outcomes, bone metastasis

## Abstract

**Introduction:** Radium-223 dichloride (radium-223) is a bone-targeting radioisotope therapy that aids in the survival of patients with metastatic castration-resistant prostate cancer (mCRPC) to bones. This study aimed to describe the clinical characteristics and outcomes of patients with mCRPC treated with radium-223 in a real-world setting. **Methods:** This was a retrospective study of patients with mCRPC treated with radium-223 between 2016 and 2020 at the London Health Sciences Centre in London, Canada. The baseline characteristics between the patients receiving 1–3 and 4–6 treatment cycles were compared using a two-sample t-test and Chi-square test. ANOVA was used to determine if there was a difference in each diagnostic variable per treatment cycle. Kaplan–Meier curves were generated to estimate progression-free survival (PFS) and overall survival in the patients treated with different numbers of cycles. **Results:** Fifty eligible patients were identified. The median age was 71 years (IQR: 66–76). Most patients (62%) received radium-223 beyond the third-line treatment. The mean number of radium-223 treatments was four. While 60% of the patients received 4–6 injections, 40% received 1–3 injections. Fifty-eight percent (58%) of the patients demonstrated a clinical benefit, with the remainder expressing either disease progression (28%) or stable disease (10%). The patients treated with 4–6 cycles had a delay to disease progression compared to those given 1–3 cycles of radium-223 (F_5,35_ = 10.52, *p* < 0.001). A higher alkaline phosphatase level prior to treatment was associated with a longer PFS (z_33_ = 2.362, *p* = 0.018). Treatment-related hospitalization for skeletal-related events was noted in 8% of the patients, and 14% required treatment discontinuation due to hematologic toxicity. **Conclusions:** This study confirms the safety of radium-223 in patients with mCRPC in a real-world setting. The radium-223 treatment was associated with a clinical benefit in the majority of the patients, particularly in those with higher pre-treatment serum alkaline phosphatase levels. Further studies to identify the predictive biomarkers are warranted to better guide the contemporary use of radium-223.

## 1. Introduction

Among men, prostate cancer is the third leading cause of cancer death and the most diagnosed malignancy, accounting for 26% of all male cancer diagnoses in the United States [1]. The primary therapy for localized disease consists of either surgical resection or radiation therapy, with or without androgen deprivation therapy (ADT). However, for patients who have recurrent or metastatic prostate cancer, ADT is the mainstay of treatment [2]. The suppression of testicular testosterone synthesis can be achieved through the administration of gonadotropin-releasing hormone agonists or antagonists, or by bilateral orchiectomy [3,4]. Although this is initially effective, most men will eventually develop a resistance to ADT, resulting in the progression of their disease despite castration, subsequently earning the label of castration-resistant prostate cancer. Once resistance to ADT has developed, the disease is considered castration-resistant, with the inappropriate reactivation of the androgen receptor axis, resulting in increased cell proliferation and growth [5]. This represents end-stage disease and is associated with significant morbidity and mortality [6].

Prostate cancer frequently metastasizes to bone, with bone metastases present in over 80% of patients with metastatic castration-resistant prostate cancer (mCRPC) [7]. There is predominance in areas of the axial skeleton, including the ribs, the pelvis, and the spine [8]. Bone metastases are associated with a significant increase in the risk of morbidity and mortality and increased pain and the occurrence of other skeletal-related events (SREs), such as spinal cord compression, pathological bone fractures, and the need for radiation or surgery to the bone [9]. These all result in a negative impact on the patients’ health-related quality of life and survival [10].

Until 2010, the only systemic treatment shown to improve survival in men with mCRPC was docetaxel-based chemotherapy [11]. Over the last decade, significant progress has been made in better understanding advanced prostate cancer, and several agents have been approved for the treatment of mCRPC based on the survival benefit, including sipuleucel-T, cabazitaxel, abiraterone acetate, enzalutamide, olaparib and radium-223. These agents are approved for specific indications in mCRPC, minimally symptomatic disease (sipuleucel-T), prior to the use of docetaxel (abiraterone and enzalutamide), post the use of docetaxel (abiraterone, enzalutamide, and cabazitaxel), and for patients whose tumors harbor a DNA deficiency repair mutation (olaparib and rucaparib) [12,13,14,15,16,17,18]. Radium-223 has been approved for use in patients with mCRPC with symptomatic bone metastases and without visceral metastases irrespective of previous docetaxel administration [19].

The radiopharmaceutical agent radium-223 dichloride is a novel, short-range targeted α-emitter that preferentially accumulates and selectively binds to areas of bone turnover, such as bone metastases. The emitted α-particle subsequently induces irreparable double-stranded DNA breaks within a short-range distance (<100 um), resulting in targeted cytotoxic activity that spares the surrounding tissue [20]. Based on the results of a phase III ALSYMPCA trial, radium-223 was proven to extend the survival and delay the time to first symptomatic skeletal events (SSEs) in patients with castration-resistant prostate cancer and symptomatic bone metastases, without visceral metastasis, irrespective of previous docetaxel use [15]. Importantly, men who underwent the radium-223 treatment did not experience significantly more grade three or four adverse events and had a meaningful improvement in their quality of life [15]. Many patients with prostate cancer are elderly, with a number of comorbidities, and the use of chemotherapy can put them at risk of toxicities or complications. Radium-223 offers an attractive alternative option to such patients to delay or avoid the need for chemotherapy, as it tends to be well tolerated. However, it does not treat non-osseous metastatic sites.

Although the previous studies have assessed the clinical outcomes in patients who received six cycles of radium-223, there is limited information of the outcomes in patients receiving fewer treatment infusions. The purpose of this study was to describe the clinical characteristics, toxicities, and outcomes of men treated with radium-223 for mCRPC at our center. We also correlated the clinical factors with the side effects and the number of cycles of radium-223, as well as treatment efficacy. Finally, we aimed to identify several clinical factors that may inform patient selection for the radium-223 treatment of mCRPC.

## 2. Methods

### 2.1. Study Design

This was a retrospective study of patients treated in the London Health Sciences Centre in London, Ontario, Canada. The eligible men had mCRPC with bone-predominant metastatic disease and received at least one radium-223 injection between 2016 and 30 April 2020. Medical oncologists obtained informed consent for and ordered the radium-223 treatments as mandated by the provincial funding guidelines. Treatment administration was conducted by Nuclear Medicine physicians. Radium-223 was given at a dose of 55 kBq/kg intravenously every 4 weeks for up to 6 total treatments provided adequate blood counts were found 7–10 days prior to administration.

With appropriate ethics approval attained (UWO HSREB#: 116200), retrospective, de-identified demographic and clinical variables, including age at radium-223 treatment initiation, Eastern Cooperative Oncology Group (ECOG) performance status, the number of radium-223 cycles received, the number of bone metastases, bisphosphonate use, the occurrence of skeletal-related events, and the transfusion of blood products, were collected. Clinical and laboratory data, including serum prostatic-specific antigen (PSA), testosterone, alkaline phosphatase, calcium, lactate dehydrogenase (LDH), hemoglobin, platelet, and neutrophil contents, were also collected. Information regarding additional treatments, including prior radiation, prior therapies, and subsequent therapies after radium-223 administration, and the occurrence and type of skeletal-related events were also recorded. Clinical outcomes were obtained from electronic medical records, with identifiers and dates removed prior to data aggregation.

Radiologic tumor assessment for each patient included computed tomography (CT) scans and bone scintigraphy performed according to the international guidelines, unless otherwise clinically indicated. The characteristics of the living patients with a clinical benefit by investigator assessment were then evaluated to identify the factors associated with subsequent progression and survival outcomes. A clinical benefit was defined by pain relief, PSA response, or alkaline phosphatase level decline.

### 2.2. Data Source

Data were collected from the patients diagnosed with bone-predominant mCRPC treated at the London Health Sciences Centre in London, Ontario, Canada, from 2016 to 2020 inclusive. The patients with the involvement of non-regional lymphadenopathy of more than 3 cm in the short-axis diameter or with visceral metastasis were excluded. Local institutional review board approval, including a waiver of consent, was obtained using a master data collection protocol.

### 2.3. Statistical Analysis

Baseline characteristics: The baseline characteristics between the patients receiving 1–3 and 4–6 treatment cycles were compared using two sample t-tests, chi-square tests, or Fisher’s Exact tests, as appropriate. A Shapiro–Wilk test was performed to assess data normality, revealing that all the variables, except age and hemoglobin level, significantly deviated from the normal distribution.

Analysis of laboratory and clinical parameters: Spearman’s correlation analysis was conducted to evaluate the relationship between pre-treatment and post-1-cycle treatment values for alkaline phosphatase (U/L), prostate-specific antigens (ng/L), hemoglobin (g/L), and platelets (×10^9^). This correlation analysis aimed to assess the changes in key patient variables after the first treatment cycle to identify the initial treatment effects and to maintain a standardized response across all the patients.

Comparative analysis of diagnostic variables across treatment cycles: ANOVA was performed to compare the diagnostic variables across different treatment cycles. This analysis examined the effects of treatment cycles, time, and their interaction to determine how these variables change over time and in response to varying treatment intensities. To evaluate the differences in time to progression across treatment regimens, Tukey’s Honest Significant Difference (HSD) test was conducted. This test provided pairwise comparisons between the different cycles of Radium-223 treatment, adjusted for multiple comparisons.

Assessment of factors influencing time to progression: To investigate the potential factors influencing the time to disease progression, Cox proportional hazard regression models were created. Each potential covariate, including demographic data, baseline clinical characteristics, and treatment-related variables, was analyzed in univariate models to identify the significant predictors of time to progression. Additionally, the data on previous therapy type and previous radiation exposure were collected for each patient to understand the impact of prior treatments on the clinical outcomes. Variables with *p*-values less than 0.05 were subsequently included in the multivariate model, allowing for the identification of independent predictors.

Kaplan–Meier Survival Analysis: Kaplan–Meier survival curves were used to estimate progression-free survival (PFS) and overall survival (OS). PFS was defined as the time for the start of treatment to disease progression (as assessed by investigators), or death from any cause, whichever occurred first. OS was defined as the time from the start of treatment until death from any cause, with censoring at the last follow-up visit if no event had occurred. A log-rank test was used to compare survival curves between the different treatments.

All statistical analyses were conducted in R Studio (v2023.03.0 + 386). All statistical tests were one-sided and considered significant at *p* < 0.05.

## 3. Results

### 3.1. Patients’ Baseline and Clinical Characteristics

Fifty patients treated with radium-223 were included. The demographic and other patient characteristics are summarized in Table 1. The median age at the initiation of the radium-223 treatment was 71 years (IQR: 66–76), and no significant difference was found between the 1–3 and 4–6 cycles of radium-223. The mean baseline PSA, alkaline phosphate, and hemoglobin values were 232.9 μg/L, 335.2 U/L, and 117.7 g/L, respectively (Table 1).

### 3.2. Treatment Pattern

Overall, 60% (30/50) received 4–6 cycles of radium-223, and 40% (20/50) of the patients received 1–3 cycles (Table 2). Thirty-one patients (62%) received radium-223 after at least three prior lines of mCRPC treatment. The other lines of systemic therapy were given either before or after radium-223 therapy. Overall, while 50% of the patients had docetaxel and abiraterone at some point in their course of the treatment; enzalutamide was given to 58% (29/50). For all the 25 patients treated with docetaxel, radium-223 was given after chemotherapy. Palliative radiation therapy was received by 11 patients (22%) (Table 3). Fourteen patients (28%) had other types of cytotoxic chemotherapy after radium-223 administration. There was no significant difference in the number of radium-223 doses completed by the patients receiving treatment before or after chemotherapy (39% vs. 32% respectively received 5–6 radium-223 doses [t_36_ = −0.60, *p* = 0.55]).

### 3.3. Characteristic Responses and Post-Progression Course

A clinical benefit from radium-223 was determined as a per patient-reported pain response, PSA response, or alkaline phosphatase level decline, following treatment initiation (Table 3). Over half of the patients demonstrated a clinical benefit (29/50, 58%), with the remaining participants found to have either disease progression (14/50, 28.0%), or stable disease (5/50, 10.0%). Treatment-related hospitalization for skeletal-related events (SREs) was noted in 8.0% (4/50) of the patients, and 14.0% (7/50) required treatment discontinuation due to prolonged myelosuppression or symptomatic anemia.

### 3.4. Predictors of Clinical Benefit and Survival

**Figure 1:**
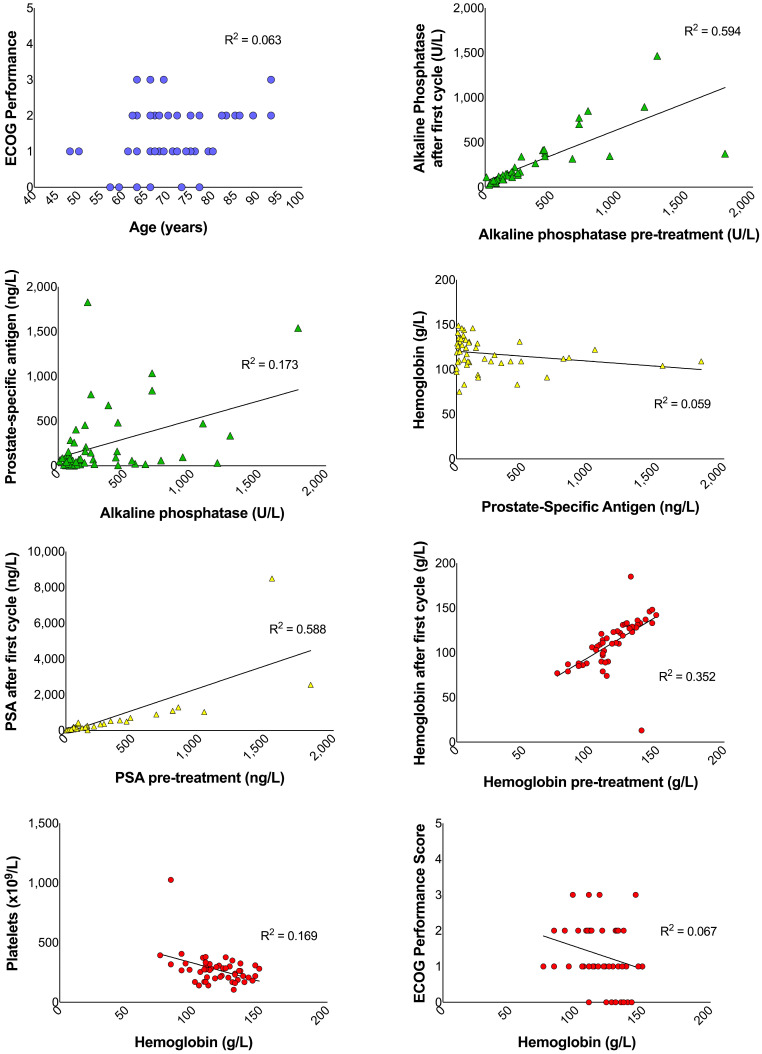
Correlations between patient variables before and after first radium-223 treatment cycles, where significant correlations are indicated by trendline (*p* < 0.05).

The alkaline phosphatase, PSA, hemoglobin, and platelet contents before and after the first cycle of treatment were positively correlated (r_49_ = 0.941, *p* < 0.001; r_49_ = 0.932, *p* < 0.001; r_49_ = 0.796, *p* < 0.001; r_49_ = 0.702, *p* < 0.001, respectively) (Table 4) (Figure 1). The ECOG performance score and the number of bone metastases were not found to be significantly correlated (r_49_ = 0.150, *p* = 0.342).

Univariate Cox proportion hazards analysis identified bone metastases (z_33_ = 2.13, *p* = 0.033), pre-treatment, and the alkaline phosphatase level (z_40_ = 3.02, *p* = 0.003) as predictors of PFS.

According to multivariate analysis, alkaline phosphatase made the most significant contribution to the difference (z_33_ = 2.362, *p* = 0.018). The number of bone metastases made a smaller contribution to the difference in the hazard ratio after adjusting for the alkaline phosphatase values (z_33_ = 1.078, *p* = 0.281).

### 3.5. Relation between Number of Radium-223 Doses/Cycles and Efficacy

Univariate analyses using the Cox proportional hazards regression models indicated more bone metastases (z_33_ = 2.13, *p* = 0.033) and higher alkaline phosphatase levels prior to treatment (z_40_ = 3.02, *p* = 0.003) were associated with an increased risk of progression for all the patients receiving treatment (Table 5). Given the effect of both the variables on PFS, a multivariate Cox proportional hazard regression model was run. When adjusting for both the factors, the effect of alkaline phosphatase prior to treatment remained significant (z_33_ = 2.362, *p* = 0.018).

The patients treated with 1–3 cycles had higher pre-treatment alkaline phosphatase (F_1,167_ = 10.53, *p* < 0.01) and PSA (F_1,180_ = 6.12, *p* = 0.01) levels compared to those receiving 4–6 cycles (Table 1).

In analysis comparing the patients who received 1–3 versus 4–6 cycles, there was a significant effect of treatment on the alkaline phosphatase, PSA, and hemoglobin contents post-cycle. The patients in the 1–3-cycle treatment group had higher alkaline phosphatase (F_1,167_ = 4.34, *p* = 0.04) and PSA (F_1,180_ = 15.84, *p* < 0.01) levels compared to those in the 4–6-cycle treatment group. The hemoglobin levels were significantly lower in the patients in the 4–6-cycle treatment group compared to those in the 1–3-cycle treatment group (F_1,193_ = 4.16, *p* = 0.04) (Table 6). There was a significant effect of time/number of cycles on the platelets, where regardless of the treatment group, the platelets declined with an increasing number of cycles (F_1,193_ = 4.45, *p* = 0.04). These data suggest that the group of patients treated with fewer cycles (1–3 cycles) had a higher disease burden with higher levels of alkaline phosphatase and PSA than the patients treated with 4–6 cycles of radium-223. In addition, with the increased number of cycles of radium-223, the levels of the platelets were lower, which indicates more bone marrow suppression due to treatment toxicity.

**Figure 2:**
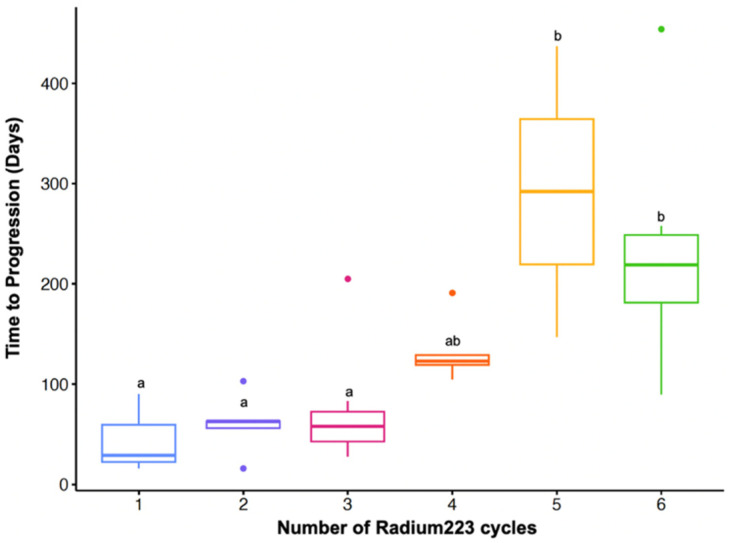
ANOVA analysis to determine statistical significance in time to progression between patients treated with different numbers of cycles of radium-223. Boxplot includes the distribution of time to progression per number of treatment cycles. Outliers are indicated with a dot, but they were not removed per intent to treat. Different letters indicate statistically significant differences (*p* < 0.05).

The median time to first progression for the overall patient cohort was 3.4 months, and the time to second progression was 6.1 months. The patients who received 1–3 cycles of radium-223 had a median time to first progression of 1.9 months, while the patients who received 4–6 cycles of radium-223 had a median time to first progression of 4.3 months (Figure 2). The median survival time in the overall patient cohort was 12.6 months, with the patients receiving 1–3 cycles of radium-223 at 9 months and 4–6 cycles at 10.8 months.

**Figure 3:**
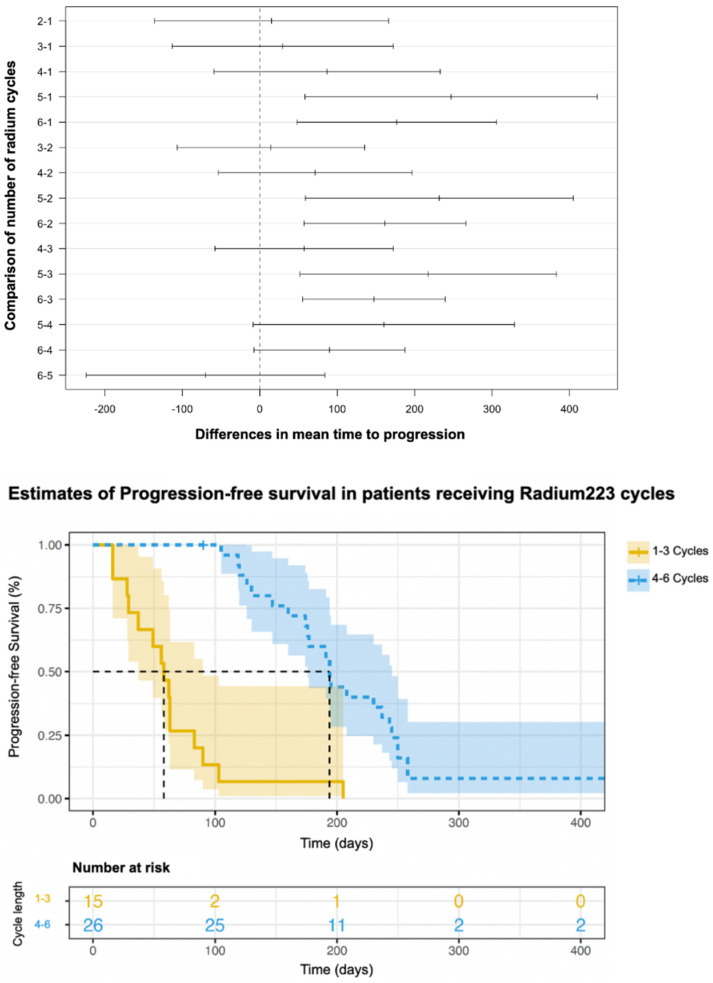
Kaplan–Meier estimates of progression-free survival of patients who underwent 1–3 cycles or 4–6 cycles.

**Figure 4:**
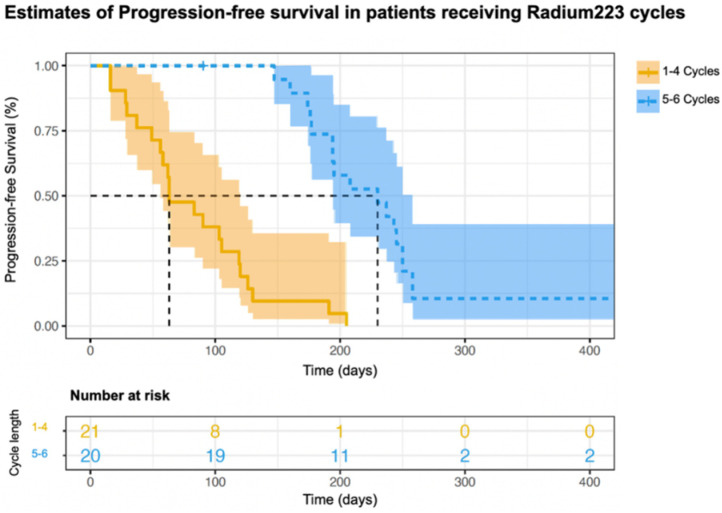
Kaplan–Meier estimates of progression-free survival of patients who underwent 1–4 cycles or 5–6 cycles.

The PFS for the patients treated with 1–3 cycles or 4–6 cycles is shown in Figure 3. A log-rank test was run to determine if there were differences in the survival distribution for the different number of cycles: 1–3 cycles of radium-223 and 4–6 cycles of radium-223. The survival distributions for the two treatment categories were significantly different (*X*^2^(1) = 36.8, *p* < 0.001). The times to progression or death for the patients treated with 5–6 cycles of treatment compared to those treated with 1–4 were significantly different (*X*^2^(1) = 35.2, *p* < 0.001) (Figure 4). These data suggest that the patients treated with more cycles of radium-223 had a better PFS.

**Figure 5:**
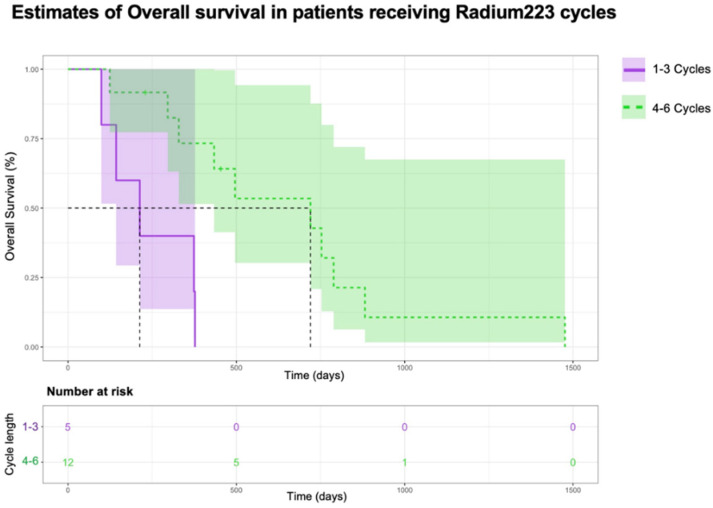
Kaplan–Meier estimates of overall survival of patients receiving 1–3 cycles or 4–6 cycles.

**Figure 6:**
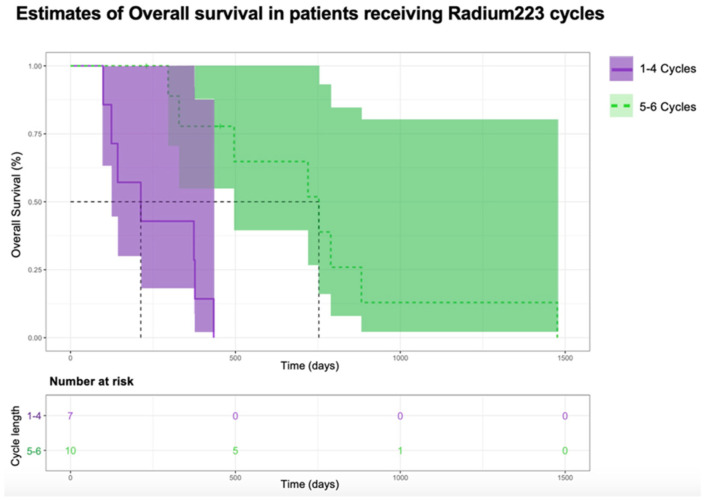
Kaplan–Meier estimates of overall survival of patients receiving 1–4 cycles or 5–6 cycles.

Overall survival was worse in the patients receiving 1–3 cycles compared to those receiving 4–6 cycles of radium-223 (*X*^2^(1) = 8.4, *p* = 0.004) (Figure 5). This was also observed with the patients receiving 1–4 cycles compared to those receiving 5–6 cycles of radium-223 (*X*^2^(1) = 11, *p* ≤ 0.001) (Figure 6).

With regards to the time to disease progression, the patients treated with 4–6 cycles of radium-223 had a longer time to disease progression compared to those treated with 1–3 cycles of radium-223 (Table 7). An ANOVA test determined that there was a statistically significant difference in the time to progression between the patients treated with different numbers of cycles of radium-223 (F_5,35_ = 10.52, *p* < 0.001).

Below, to further explain Table 7, we show the differences in the mean time to progression regarding the number of radium-223 treatment cycles.

## 4. Discussion

This retrospective study of patients with bone-predominant mCRPC highlights the real-world utilization of radium-223. In our study, most patients received fewer than the recommended six cycles of radium-223. Most patients receiving radium-223 had significant prior systemic therapy, with the administration of a third-line therapy in 12%, and that of a fourth-line therapy or greater in 62% of the patients, respectively. Over half of the patients were felt to have experienced a clinical benefit, and only 14% of the patients requiring treatment discontinuation due to hematologic toxicity.

Our study attempted to identify the clinical and/or laboratory markers associated with responses and survival outcomes. Both a higher serum alkaline phosphatase level pre-treatment and a higher number of bone metastases appeared to be associated with a higher risk of disease progression, consistent with the hypothesis of a higher disease burden. The treatment landscape of metastatic CRPC has evolved in the last decade. As a result of the complexity of treatment options, there is a need to improve and find new biomarkers to potentially better characterize those patients with clinical benefits and guide and individualize treatment selection. As per the guidelines, the performance status, pain improvement, anemia, prostate-specific antigen kinetics, and the markers of bone metabolism (urinary N-telopeptide and bone-specific alkaline phosphatase) have been used as prognostic markers. Alkaline phosphatase (ALP) is a byproduct of osteoblast activity, and a high serum level of ALP indicates increased bone turnover and active bone formation [21]. Serum alkaline phosphatase was found to be significantly associated with a worse survival rate in prostate cancer [22]. In clinical practice, the careful selection of patients to undergo treatment with radium-223 should be highly considered to find those who might benefit from more treatment. Given the results of our study, serum alkaline phosphatase should be better explored as a potential prognostic marker.

In the ALSYMPCA trial [15], 58% of the patients received all the six injections of radium-223 compared to 42% in our cohort. This difference may be explained by the fact that the results of phase three clinical trials do not always reflect the same patient characteristics and outcomes as those treated in the real world. Our retrospective study also aimed to analyze if there were any differences in the patients treated with 1–3 versus 4–6 radium-223 treatments. The patients treated with 1–3 cycles of radium-223 had higher pre-treatment serum alkaline phosphatase and PSA levels and lower hemoglobin levels compared to those of the patients treated with 4–6 cycles of radium-223. The patients who received 1–3 cycles of radium-223 had an inferior median time to first progression (1.9 months) versus those treated with 4–6 cycles (4.3 months). Similarly, the overall survival was worse for the group of patients receiving 1–3 cycles of radium-223 (9 months) in comparison to the overall survival of those who received 4–6 cycles (8.8 months).

More recently, PEACE-3 trial, presented at the European Society for Medical Oncology (ESMO) Congress 2024, showed both radiographic progression-free survival and overall survival benefits with the combination of radium 223 and enzalutamide in first line setting for mCRPC. These findings also raise a question about the best timing of radium 223 in the therapeutic sequence and could add this as another area for investigation [23].

Our study has a number of limitations. First, the retrospective nature of this study limits the assessment of benefits and toxicity. Second, it reflects the experience at a single academic institution prior to the COVID-19 pandemic. Finally, overall survival analysis did not adjust for the use of other mCRPC life-prolonging therapies.

In conclusion, our study provides evidence that radium-223 treatment was effective and safe in patients with mCRPC in a real-world setting at an academic clinical practice. We have identified clinical factors potentially influencing the treatment benefits and cancer control. The optimal timing and sequencing of radium-223 in the expanding systemic therapy armamentarium for mCRPC remains to be defined. A better understanding of CRPC biology and the identification of predictive biomarkers for radium-223 benefits and additional prospective studies will be important to optimize the clinical use of radium-223.

## Figures and Tables

**Table 1:** Exploratory statistics.

Variable	Mean Value ± SE	Statistical Difference
Age (years) 1–3 cycles 4–6 cycles	71.18 ± 1.34 70.15 ± 1.81 71.87 ± 1.89	t_46_ = 0.66, *p* = 0.52
Clinical stage	4 ± 0	
ECOG Performance overall 1–3 cycles 4–6 cycles	1.32 ± 0.12 1.55 ± 0.18 1.17 ± 0.15	t_41_ = 1.6, *p* = 0.17
Time to progression (weeks) 1–3 cycles 4–6 cycles	154.29 ± 15.86 63.87 ± 12.08 206.46 ± 17.02	t_39_ = 6.83, *p* < 0.001
Number of bone metastases 1–3 cycles 4–6 cycles	8.52 ± 0.34 9.21 ± 0.39 7.96 ± 0.51	t_38_ = 1.96, *p* = 0.06
Number of blood transfusions	0.76 ± 0.17	
Number of radium-223 cycles	4.14 ± 0.26	
Alkaline Phosphatase prior to treatment (U/L) 1–3 cycles 4–6 cycles	335.22 ± 53.38 537.95 ± 102.99 200.07 ± 42.61	t_25_ = 3.03, *p* < 0.01
Prostate-specific antigen prior to treatment (ng/L) 1–3 cycles 4–6 cycles	232.88 ± 54.18 362.44 ± 93.50 146.52 ± 61.67	t_34_ = 1.93, *p* = 0.06
Hemoglobin prior to treatment (g/L) 1–3 cycles 4–6 cycles	117.7 ± 2.48 114 ± 3.35 120.17 ± 3.45	t_46_ = 1.28, *p* = 0.21
Platelets prior to treatment (×10^9^/L) 1–3 cycles 4–6 cycles	273.3 ± 18.42 254.3 ± 17.62 285.97 ± 28.39	t_45_ = 0.95, *p* = 0.35

**Table 2:** Radium-223 cycle treatment pattern.

Characteristics		Total
Number of Radium-223 cycles, n (%) (n = 50)	1	4 (8.0%)
	2	9 (18.0%)
	3	7 (14.0%)
	4	7 (14.0%)
	5	2 (4.0%)
	6	21 (42.0%)
Radium-223 line of treatment, n (%)	First	1 (2.0%)
	Second	19 (38.0%)
	Third	20 (40.0%)
	Fourth	9 (18.0%)
	Fifth	1 (2.0%)

**Table 3:** Patients’ characteristic responses and post-progression course.

Characteristics		Total
Clinical benefit, n (%) (n = 50)	Yes	29 (58.0%)
	Progressive disease	14 (28%)
	Stable disease	5 (10.0%)
	Unknown	2 (4.0%)
Other lines of treatment, n (%) (n = 50)	Docetaxel	25 (50.0%)
	Cabazitaxel	2 (4.0%)
	Enzalutamide	29 (58.0%)
	Abiraterone	25 (50.0%)
	Carboplatin-Epirubicin	1 (2.0%)
	Clinical Trial	3 (6.0%)
	Palliative radiation	11 (22.0%)
	Other	3 (6.0%)
Skeletal related events (SRE) n (%)	No	46 (92%)
Type of SREs, n (%)	Spinal Cord Compression	3 (6.0%)
	Pathologic Fracture	1 (2.0%)
	Hypercalcemia	0 (0.0%)
	Surgery or radiation required	4 (8.0%)
Radium-223 discontinuation due myelosuppression, n (%)		9 (14.0%)

**Table 4:** Spearman’s correlation values reflecting correlation between patient variables before and after first treatment cycle of radium-223. (**A**) Comparison of ALP, PSA, hemoglobin, and platelet contents. (**B**) Comparison of patients’ age and ECOG status.

(**A**)						
		**Alkaline Phosphatase**	**Prostate-Specific Antigen**	**Hemoglobin**	**Platelets**	
**Alkaline phosphatase**			***r_49_ = 0.371,*** ***p = 0.008***	***r_49_ = 0.371,*** ***p = 0.008***	r_49_ = −0.105, *p* = 0.467	
**Prostate-specific antigen**		***r_49_ = 0.371,*** ***p = 0.008***		*r_49_ = −0.290,* *p = 0.041*	r_49_ = 0.189, *p* = 0.189	
**Hemoglobin**		***r_49_ = 0.371,*** ***p = 0.008***	*r_49_ = −0.290,* *p = 0.041*		***r_49_ = −0.309,*** ***p = 0.028***	
**Platelets**		r_49_ = −0.105, *p* = 0.467	r_49_ = 0.189, *p* = 0.189	***r_49_ = −0.309,*** ***p = 0.028***		
(**B**)						
	**Age**	**ECOG**	**Alkaline Phosphatase**	**Prostate-Specific Antigen**	**Hemoglobin**	**Platelets**
**Age**		r_49_ = 0.250, *p* = 0.079	r_49_ = 0.220, *p* = 0.125	r_49_ = −0.045, *p* = 0.756).	r_49_ = 0.021, *p* = 0.886	r_49_ = −0.212, *p* = 0.139
**ECOG**	r_49_ = 0.250, *p* = 0.079		r_49_ = 0.101, *p* = 0.483	r_49_ = −0.083, *p* = 0.566	***r_49_ = −0.306,*** ***p = 0.031***	r_49_ = −0.021, *p* = 0.884

**Table 5:** Cox proportion hazard result of each potential covariate that was considered to potentially impact time to progression. Covariates with statistically significant effects on time to progression are bolded (*p* < 0.05).

Covariate	Cox Proportion Hazard Test Result
Age	z_40_ = 0.023, *p* = 0.982
Number of bone metastases	**z_33_ = 2.13, *p* = 0.033**
Previous therapy type	z_40_ = −0.234, *p* = 0.815
Previous radiation	z_40_ = 1.13, *p* = 0.258
ECOG performance score	z_40_ = 1.21, *p* = 0.228
Number of blood transfusions	z_40_ = 0.407, *p* = 0.684
Alkaline phosphatase prior to treatment	**z_40_ = 3.02, *p* = 0.003**
Prostate-specific antigen prior to treatment	z_40_ = 1.61, *p* = 0.108
Hemoglobin prior to treatment	z_40_ = −0.53, *p* = 0.596
Platelets prior to treatment	z_40_ = 0.541, *p* = 0.589

**Table 6:** ANOVA analysis comparing diagnostic variables across treatment cycles. This analysis investigated effects of time, treatment, and their interaction to understand how these variables change over time and in response to different treatment regimens. Bold values indicate statistically significant differences (*p* < 0.05) in how treatment intensity influences clinical metrics.

	1–3 vs. 4–6 Cycles			1–3 vs. 4–6 Cycles		
	Time	Treatment	Time × Treatment	Time	Treatment	Time × Treatment
**Alkaline phosphatase**	F_1,167_ = 0.24 *p* = 0.63	**F_1,167_ = 10.53** ***p* < 0.01**	F_1,167_ = 0.16 *p* = 0.69	F_1,167_ = 0.001 *p* = 0.97	**F_1,167_ = 4.34** ***p* = 0.04**	F_1,167_ = 2.06 *p* = 0.15
**Prostate-specific antigen**	F_1,180_ = 0.01 *p* = 0.92	**F_1,180_ = 6.12** ***p* = 0.01**	F_1,180_ = 2.18 *p* = 0.14	F_1,180_ = 0.43 *p* = 0.51	**F_1,180_ = 15.84** ***p <* 0.01**	F_1,180_ = 0.04 *p* = 0.84
**Hemoglobin**	F_1,193_ = 0.58 *p* = 0.45	F_1,193_ = 1.57 *p* = 0.21	F_1,193_ = 0.04 *p* = 0.84	F_1,193_ = 1.35 *p* = 0.25	**F_1,193_ = 4.16** ***p* = 0.04**	F_1,193_ = 0.11 *p* = 0.75
**Platelets**	**F_1,193_ = 4.05** ***p* = 0.05**	F_1,193_ = 0.41 *p* = 0.52	F_1,193_ = 0.01 *p* = 0.92	**F_1,193_ = 4.45** ***p* = 0.04**	F_1,193_ = 0.58 *p* = 0.45	F_1,193_ = 0.75 *p* = 0.39

**Table 7:** Tukey’s HSD test comparing time to progression between patients treated with different cycles of radium-223. This test was chosen to evaluate pairwise differences in time to progression across treatments, while adjusting for multiple comparisons. Doing so provides insight into how increasing number of treatment cycles impacts progression of disease. Statistically significant comparisons (*p* < 0.05; bolded) indicate differences in time to progression given number of radium-223 cycles patients received. These findings suggest that increasing treatment cycles may delay disease progression.

	1 Cycle	2 Cycles	3 Cycles	4 Cycles	5 Cycles	6 Cycles
**1 cycle**		*p* = 0.999, 95% C.I. = [−135.8, 166.2]	*p* = 0.988, 95% C.I. = [−113.1, 172.2]	*p* = 0.485, 95% C.I. = [−59.4, 233.0]	***p = 0.005, 95% C.I. = [58.3, 435.7]***	***p = 0.003, 95% C.I. = [47.9, 305.8]***
**2 cycles**	*p* = 0.999, 95% C.I. = [−135.8, 166.2]		*p* = 0.999, 95% C.I. = [−106.7, 135.4]	*p* = 0.526, 95% C.I. = [−53.6, 196.8]	***p* = 0.004, 95% C.I. = [58.8, 404.8]**	***p = 0.001, 95% C.I. = [57.1, 266.1]***
**3 cycles**	*p* = 0.988, 95% C.I. = [−113.1, 172.2]	*p* = 0.999, 95% C.I. = [−106.7, 135.4]		*p* = 0.666, 95% C.I. = [−57.8, 172.3]	***p* = 0.004, 95% C.I. = [51.7, 383.2]**	***p = 0.0003, 95% C.I. = [55.2, 239.3]***
**4 cycles**	*p* = 0.485, 95% C.I. = [−59.4, 233.0]	*p* = 0.526, 95% C.I. = [−53.6, 196.8]	*p* = 0.666, 95% C.I. = [−57.8, 172.3]		*p* = 0.071, 95% C.I. = [−8.6, 329.0]	*p* = 0.084, 95% C.I. = [−7.4, 187.5]
**5 cycles**	***p* = 0.005, 95% C.I. = [58.3, 435.7]**	***p* = 0.004, 95% C.I. = [58.8, 404.8]**	***p* = 0.004, 95% C.I. = [51.7, 383.2]**	*p* = 0.071, 95% C.I. = [−8.6, 329.0]		*p* = 0.743, 95% C.I. = [−224.3, 83.9]
**6 cycles**	***p* = 0.003, 95% C.I. = [47.9, 305.8]**	***p* = 0.0006, 95% C.I. = [57.1, 266.1]**	***p* = 0.0003, 95% C.I. = [55.2, 239.3]**	*p* = 0.084, 95% C.I. = [−7.4, 187.5]	*p* = 0.743, 95% C.I. = [−224.3, 83.9]	

## Data Availability

All the data generated or analyzed during this study are included in this article.
